# Clinical, Radiological and Functional Characteristics of Pulmonary Diseases among HTLV-1 Infected Patients without Prior Active Tuberculosis Infection

**DOI:** 10.3390/pathogens10070895

**Published:** 2021-07-14

**Authors:** Rodrigo Cachay, Marcela Gil-Zacarias, Takashi Watanabe-Tejada, Alvaro Schwalb, Fernando Mejía, Oscar Gayoso, Eduardo Gotuzzo

**Affiliations:** 1 Instituto de Medicina Tropical Alexander von Humboldt, Universidad Peruana Cayetano Heredia, Lima 15102, Peru; marcela.gil.z@upch.pe (M.G.-Z.); martin.watanabe.t@upch.pe (T.W.-T.); alvaro.schwalb@upch.pe (A.S.); fernando.mejia.c@upch.pe (F.M.); oscar.gayoso.c@gmail.com (O.G.); eduardo.gotuzzo@upch.pe (E.G.); 2 Alberto Hurtado School of Medicine, Universidad Peruana Cayetano Heredia, Lima 15102, Peru; 3 Infectious, Tropical and Dermatological Diseases, Hospital Cayetano Heredia, Lima 15102, Peru; 4 Pulmonology Service, Hospital Cayetano Heredia, Lima 15102, Peru

**Keywords:** HTLV-1, pulmonary disease, pulmonary function, epidemiology

## Abstract

The lifelong infection with the human T lymphotropic virus type 1 (HTLV-1) has been associated with a variety of clinical manifestations; one of the less-explored is HTLV-1-associated pulmonary disease. Imaging of lung damage caused by the HTLV-1 hyperinflammatory cascade can be similar to sequelae from TB infection. Our study aims to describe the pulmonary lesions of HTLV-1-positive patients without past or current active TB and evaluate pulmonary function. We found that nine out of fourteen patients with no known TB disease history presented bronchiectasis, mainly found bilaterally while five presented pulmonary fibrosis. A normal pattern was found in most patients with a pulmonary functional test. Furthermore, there was no association between the PVL and the chest-CT scan findings, nor with spirometry results. However, the sample size was insufficient to conclude it.

## 1. Introduction

The lifelong infection with the human T lymphotropic virus type 1 (HTLV-1) has been associated with a variety of clinical manifestations including adult T cell leukemia/lymphoma (ATLL), HTLV-1 associated myelopathy/tropical spastic paraparesis (HAM/TSP), uveitis, Sjögren’s syndrome, and others [1]. A less-explored manifestation, bronchiectasis, has been consistently reported among HTLV-1-positive patients from Australia, Japan, United Kingdom, and Brazil [2,3,4]. Other chronic lung diseases such as bronchitis and pulmonary fibrosis have also been described in HTLV-1 carriers [4,5,6,7]. These lesions can occur in symptomatic patients and less frequently in asymptomatic patients; however, this has hardly been recognized [8,9]. While these lesions arise due to an exaggerated immune response to the virus, the acquired immunodeficiency conveys a higher risk of opportunistic infections such as cryptococcosis and tuberculosis (TB), developing severe forms of these diseases [4,10]. 

In particular, the coinfection of TB and HTLV-1 should be taken into account given its high prevalence in areas where both diseases are endemic, the increased likelihood of hospitalization, and the high mortality rates among co-infected individuals [11,12,13,14,15]. Persistent pulmonary lesions occur in up to half of active TB survivors despite adequate treatment [16]. TB lesions may encompass cavitation, fibrosis, or bronchiectasis [17]. Imaging of pulmonary sequelae from TB disease can be similar to those caused by the hyper-inflammatory cascade of HTLV-1, usually described as peripheral lung parenchymal lesions mainly centrilobular nodules, ground-glass opacities, and bronchiectasis [3]. This hypothesizes the need to consider HTLV-1 in the differential diagnosis. 

Peru is a TB-endemic country with an estimated incidence of 119 cases per 100,000 inhabitants [18]. Additionally, the prevalence of HTLV-1 has been estimated between 1–2% of the Peruvian population [19]. The co-infection of HTLV-1 with TB has been estimated between 2.8% to 5.6% among drug-susceptible and multidrug-resistant TB, respectively [13,20]. Few studies have characterized the lung lesions of HTLV-1 infected patients amongst those without known TB history. The study aimed to describe the pulmonary lesions and evaluate pulmonary function of HTLV-1-positive patients without past or current active TB disease.

## 2. Results

From 3500 patients registered in the HTLV-1 clinical cohort database, 53 met the inclusion criteria; nonetheless, only 14 patients (26%) were included in the study (Appendix A). Among these, eight (57%) were female and the median age was 60.6 years old (IQR: 52–69). Although six patients were originally from the Peruvian highlands, 13 were currently living in Lima, a coastal region. Four patients were obese (BMI ≥ 30 kg/m^2^) and one was overweight (BMI ≥ 25–29.9 kg/m^2^); median BMI was 28 kg/m^2^. Other comorbidities are listed in Table 1. Five presented at least one HTLV-associated disease, these are described in Table 2.

Regarding risk factors associated with pulmonary disease, six patients reported daily exposure to wood-burning stoves for an average of 24 years (0.2–70 years), five were active smokers, and another four reported exposures to secondhand smoke. Three patients had occupational exposure to construction, carpentry, and cooking fumes. Likewise, two patients reported long-term use of losartan and one reported the use of sertraline. Additionally, five patients reported at least one episode of wheezing cough during their adulthood (≥18 years old) and only one had episodes of wheezing in childhood, although none have been previously diagnosed with asthma. Eight patients reported gastroesophageal reflux symptoms; three had allergic rhinitis and five patients reported chronic dry cough. Six participants reported between 2–5 respiratory infections per year, with none requiring hospitalization. Previous influenza and pneumococcal vaccination was reported in seven and three patients, respectively.

Only 12 patients were evaluated by a pulmonologist. Five patients had active cough; five with productive cough, two with purulent sputum based on SCC, and two reported hemoptysis. Four patients presented with active dyspnea, of whom two obtained a score of 2 on the mMRC, one had a score of 3 and only one had a score of 4. All patients were tachypneic (≥20 breaths/minute) at physical examination, with a median of 23 (IQR: 20–24); the median oxygen saturation was 94.7% (IQR: 92.6–96.8%).

**Figure 1:**
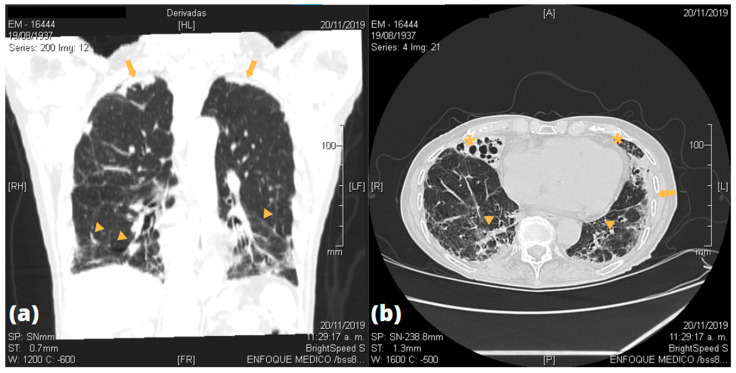
Images of an 85 years-old female patient with multiple HTLV-1 associated disorders (infective dermatitis, TSP/HAM, Sjögren’s syndrome). (**a**) Coronal CT scan shows peripheral images of ground-glass pattern in the lower lobes (arrowheads) and apical pleural thickening (arrows). (**b**) Transverse CT scan shows bilateral saccular bronchiectasis in the lower lobes (asterisks), thickening of bronchovascular bundles, and presence of ground-glass pattern (arrowheads) with pleural thickening (arrow).

**Figure 2:**
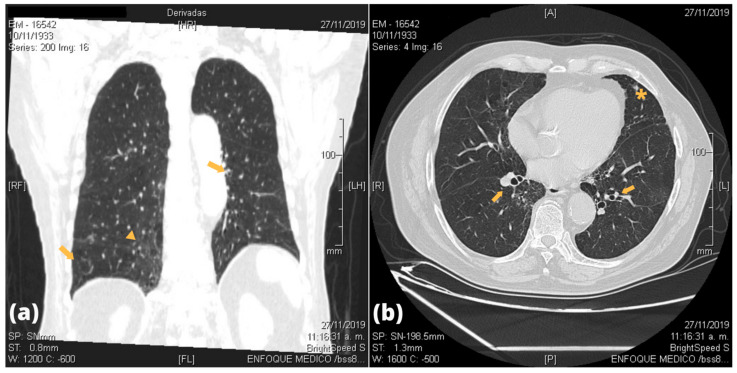
Images of an 86 years-old male patient with TSP/HAM and neurogenic bladder. (**a**) Coronal CT scan shows diffuse ground-glass pattern (arrowheads) in the right paramediastinal region, elevation of the left hemidiaphragm and presence of bronchiectasis (arrows). (**b**) Transverse CT scan shows diffuse ground-glass pattern, bronchiectasis (arrows), and presence of a nodular image (asterisk).

Imaging studies by CT scans of the lungs were done in all patients, of which twelve showed abnormal findings. Figure 1 and Figure 2 show some of the most frequent lesions found in the participants’ CT scans. Most of them were localized in the lower lobe of either lung. Bronchiectasis were present in nine patients, and five were bilateral. Ground-glass opacities (GGO) were found in seven patients, whereas bilateral pleural thickness in five patients. All lung lesions are shown in Table 3 and a detailed descriptions of radiological lung findings on chest CT scans are shown in Appendix B. Pulmonary function by spirometry was successfully assessed in 12 patients as two were unable to tolerate the test. Only two had abnormal pattern findings: one had a restrictive pattern, and the other an obstructive pattern. PVL was measured in all patients; the median PVL value was 1925 copies/mL (IQR = 981–2868 copies/mL).

Moreover, we did not find significant difference in PVL among those with a history of HTLV-associated disease (*p* = 0.22) nor in those with abnormal findings on chest-CT scan (*p* = 0.28). Likewise, there was no difference between PVL median among those with bronchiectasis (*p* = 1.0) nor pulmonary fibrosis (*p* = 0.08). Median PVL was 1925 copies/mL (IQR: 981–2868 copies/mL) and 1205 copies/mL among those with abnormal pattern on spirometry (*p* = 0.59).

## 3. Discussion

Our study aims to describe the pulmonary damage of HTLV-1 infected patients either on chest-CT scan and in the pulmonary functional tests as well as clinical characteristics of pulmonary disease. We found that most patients with no known TB disease history presented bronchiectasis (mainly found bilaterally) and pulmonary fibrosis. Nonetheless, a normal pattern was found in most patients with a pulmonary functional test. Furthermore, there was no association between the PVL and the chest CT scan findings, nor with spirometry results.

Despite it being a neglected tropical disease, the multi-systemic impact of HTLV-1 infection on the health of patients has been thoroughly studied [21]. Lung injuries due to viral damage are a less-explored spectrum in the context of HTLV-1 infection, albeit the tropism has an estimated prevalence of 30.1% among HTLV-1 carriers [9,22,23]. However, the differential diagnosis, which includes TB disease, may cause similar findings on imaging studies and therefore should be evaluated, especially in hyperendemic countries such as Peru. By excluding patients with known prior active TB disease and who had a documented lung disease, we attempted to isolate the extension of pulmonary damage caused by the virus.

Lung infection due to non-tuberculous mycobacteria (NTM) often occurs in the context of pre-existing lung or chronic diseases [24]; however, the prevalence in developing countries appears to be low compared to developed ones [25]. While the prevalence in Peru is unknown, with only eight cases reported [26,27,28], the national burden of TB disease would make lung lesions by NTM a trivial proportion.

The impact of HTLV-1 infection on pulmonary functional tests has been described as normal pattern on asymptomatic carriers, whilst abnormal findings, either restrictive or obstructive, among those with HAM/TSP, although studies are scarce [29].

The chronic hyperinflammatory cascade found in the bronchoalveolar lavage plays a critical role in the development of pulmonary changes in HTLV-1 patients, activating the recruitment and adhesion of cytokines as well as fibrosis of the lung tissue, leading to structural damage of the lungs [4]. Higher PVL is associated with lung injuries, particularly with bronchiectasis [5,7]; however, we did not find such association in our study. Bronchitis/bronchoalveolitis pattern was found in 30% of HTLV-1 patients with abnormal radiological findings in a Japanese study, whilst bronchiectasis were reported in 19.2% of Australian Aboriginal population with an increased mortality in younger ages associated with bronchiectasis complications [5,30]

While viral lung damage seems certain, there are many environmental factors that might contribute to the development of pulmonary diseases, and that are likely to be expressed in older patients. Exposure to environmental factors is a less controlled factor in studies although the impact on lung health has been well recognized and the household air pollution has been estimated as the leading environmental cause of death worldwide [31,32,33] Biomass fuel and domestic smoking such as wood-burning stoves, a common practice in the Peruvian Andean region, are a known risk factor for several pulmonary diseases including non-TB bronchiectasis, chronic bronchitis, cor pulmonale, and convey 2.5-times greater risk to develop chronic obstructive pulmonary disease [34,35,36]. Four out of the nine patients who presented bronchiectasis on chest-CT scan reported daily use of wood-burning stoves as the main source of cooking. On the other hand, work-related exposure such as pneumoconiosis and drug-related damage may lead to interstitial lung diseases [37]. We found that among those with GGO suggesting fibrotic changes on chest-CT scan, one was an active smoker, four has been daily exposed to secondhand smoke but none had been exposed to mines, paint, lead, or industrial smoke. Furthermore, none of the three patients who reported long-term use of drugs related to lung damage (losartan and sertraline) presented GGO findings on imaging.

The clinical implications of lung damage in HTLV-1 patients need further attention. Chronic cough and dyspnea were the most common symptoms found in our population and the median oxygen saturation was below the normal range. These implications should be assessed by physicians in a rehabilitation program to minimize the burden on quality of life and daily activities. Several authors have recommended lung damage screening through chest-CT scan and a detailed physical examination in HTLV-1 patients [3,7]

Our study is limited due to the small number of participants, it was only possible to evaluate less than a third of the cases identified in the cohort, and therefore any associations should be further evaluated in a larger population with adequate funding to achieve a better follow-up. Additionally, as it has been found lung damage in patients with sub-clinical tuberculosis [38], having no prior history of active TB pulmonary disease as exclusion criteria to rule out the infection and TB-related lung lesions is insufficient. All patients should undergo greater sensitive tests, such as interferon-gamma release assay or protein purified derivative skin test, to confirm the absence of TB exposure. It is necessary to evaluate the risk factors for the development of pulmonary diseases in greater detail, with our study we cannot assert that HTLV-1 infection was the only cause of the pulmonary lesions. Finally, imaging, pulmonary function tests and PVL measurements were assessed in different points from infection or symptom onset, varying greatly between patients. Far more prospective studies on the natural history of HTLV-1 are needed to understand our remaining questions.

## 4. Methods

### 4.1. Study Design and Population

We conducted a cross-sectional study between September to December 2019 among HTLV-1-infected patients. Participants were searched from the records of the HTLV-1 clinical cohort of the Instituto de Medicina Tropical Alexander von Humboldt, the largest cohort in Peru with approximately 3500 patients and relatives. The cohort database was searched for patients with (1) diagnosis of HTLV-1 infection using two enzyme-linked immunosorbent assays and/or one confirmatory test (Western blot), (2) a documented lung disease: including bronchitis, pulmonary fibrosis, bronchiectasis or asthma, and (3) without clinical history of tuberculosis infection or current active tuberculosis lung disease. History of tuberculosis disease was evaluated through a detailed review of the medical records, previous imaging results and during an interview.

### 4.2. Procedures

After providing written informed consent, all participants underwent a non-contrast chest computed tomography (CT) scan and a spirometry test. Blood samples were drawn to measure HTLV-1 proviral load by RT-PCR. Participants were then evaluated by a pulmonologist, who performed a focused physical exam and recorded the patient’s medical history, HTLV-1-asssociated comorbidities, risk factors for asthma, bronchiectasis, pulmonary fibrosis, infections and vaccination history. The Sputum Color Chart (SCC) was applied to characterize the sputum if they presented it at the time of the interview. This chart uses eight photographs of sputum of patients with bronchiectasis, and correlate bacterial colonization with three typical gradations of color (purulence): clear (mucoid), pale yellow/pale green (mucopurulent), and dark yellow/dark green (purulent) [39]. The modified Medical Research Council (mMRC) breathlessness scale was used to characterize shortness of breath by severity, from grade 0 to 4 as follows: (0) get breathless with strenuous exercise; (1) get short of breath when hurrying on level ground or walking up a slight hill; (2) need to walk slower due to breathlessness; (3) stop for breath after walking about 100 m; and (4) feel too breathless to leave the house or get dressed [40].

Imaging findings on chest CTs were classified as following: (a) bronchial lesions, (b) alveolar damage, (c) parenchymal damage and d) pleural damage. Chest-CT scans were reported by a radiologist and were later reviewed by a pulmonologist. Spirometry results were interpreted by the pulmonologist and categorized as normal, obstructive, or restrictive patterns. Spirometry measures included forced vital capacity, forced expiratory volume in one second, the FEV1/FVC ratio, forced expiratory flow at 50% and the peak expiratory flow.

### 4.3. Statistical Analyses

Participant data was anonymized and entered into a secure database. Analyses were performed using Stata SE 16.1 (StataCorp, US). A descriptive analysis was performed using percentages (%) to describe frequencies for categorical variables; median with interquartile range was used to describe numerical data. In the bivariate analysis, the chi-squared test was used for categorical variables; the Mann–Whitney U test and Kruskal–Wallis rank test were used for two or more continuous variables, respectively.

### 4.4. Ethical Aspects

The study was approved by the Institutional Ethics Committee of Universidad Peruana Cayetano Heredia (SIDISI: 104427). All participants provided written informed consent before imaging/laboratory procedures and specialist evaluation.

## Figures and Tables

**Table 1:** Sociodemographic characteristics of HTLV-1 patients with lung diseases without prior known TB disease.

Characteristics (*n* = 14)	Frequency
Female sex, (%)	8 (57)
Age, years, median (IQR)	60.6 (52–69)
BMI, kg/m^2^	
Normal (18.5–24.9)	6/11
Overweight (25–29.9)	1/11
Obese (>30)	4/11
Place of origin	
Highlands	5/14
Coast region	9/14
Place of residence	
Lima	12/14
Outside Lima	2/14
Pneumoconiosis-risk occupation	3/12
Comorbidities	4/12
Cardiovascular: high blood pressure, heart failure	4/12
Endocrine: diabetes mellitus type II, hypothyroidism	3/12
Cancer	1/12
Lung damage-associated drugs: losartan and sertraline	3/12
Previous HTLV-1 associated disease	5/12
Infective dermatitis	1/12
Tropical spastic paraparesis	4/12
Strongyloidiasis	1/12
Uveitis	1/12
Sjögren’s syndrome	1/12

HTLV-1: human T lymphotropic virus type 1; TB: tuberculosis infection; BMI: body mass index; MCTD: mixed connective tissue disorder.

**Table 2:** HTLV-1-associated disease among the patients included in the study.

Patient ID	HTLV-1 Associated Disorder
P2	HAM/TSP
P4	Strongyloidiasis
P6	Strongyloidiasis, HAM/TSP, uveitis
P11	Infective dermatitis, HAM/TSP, Sjögren’s syndrome
P15	HAM/TSP

P: patient; HTLV-1: human T lymphotropic virus type 1; HAM/TSP: HTLV-1 associated myelopathy/tropical spastic paraparesia.

**Table 3:** Radiological findings on chest-CT scan of HTLV-1 infected patients.

Radiological Lung Lesion	Frequency
Lobe compromise	
Lower lobe: RL, LF	10/14, 9/14
Medial lobe: RL	7/14
Upper lobe: RL, LF	7/14, 5/14
Bronchial lesions	
Bronchiectasis	9/14
Bilateral	5/9
Atelectasis	2/14
Alveolar damage	0/14
Parenchymal damage	
GGO/infiltrates	7/14
Fibro-retractable tracts	10/14
Bilateral pleural thickness	5/14

CT: computed tomography; HTLV-1: human T lymphotropic virus type 1; RL: right lobe; LF: left lobe; GGO: ground-glass opacities.

**Figure A1:**
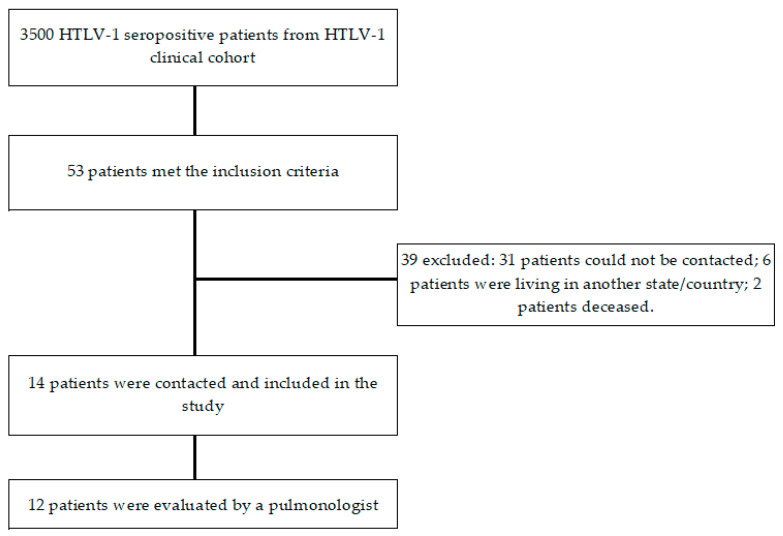
Flow chart of participants in the study.

## Data Availability

The data presented in this study are openly available in FigShare at 10.6084/m9.figshare.14602896.

## Appendix B

**Table A1:** Detailed radiological findings on chest-CT scan of HTLV-1 infected patients.

Patients	Bronchial Lesion	Alveolar Lesion	Parenchymal Lesion	Pleural Lesion
P1	None	None	Scarce right inferior fibrous sequelae tracts	None
P2	Bilateral tubular bronchiectasis	None	Bilateral GGO; scarce right inferior fibrous retractile tracts	None
P3	Bilateral varicose bronchiectasis	None	None	None
P4	None	None	Bilateral apical fibrous retractile tracts	Bilateral apical pleural thickening
P5	Right medial bronchiectasis	None	Bilateral inferior GGO; bilateral apical fibrous retractile tracts; right medial air cyst	Bilateral apical pleural thickening
P6	Right medial and left inferior bronchiectasis	None	Bilateral inferior fibrous retractile tracts; right apical interstitial infiltrate	None
P7	Left atelectasis	None	Bilateral inferior interstitial infiltrate	None
P8	Bilateral cylindrical hilio-perihiliar bronchiectasis; laminar atelectasis	None	Bilateral apical fibrous retractile tracts	Right apical pleural thickening
P9	Bilateral tubular bronchiectasis	None	Bilateral GGO with fibrous tracts	None
P11	Right medial varicose bronchiectasis	None	Bilateral inferior GGO and interlobular septal interstitial thickening; bilateral apical thick fibrous sequelae tracts	Bilateral apical pleural thickening
P12	Right tubular bronchiectasis	None	Bilateral inferior opacities and fibro retractable tracts	Bilateral pleural thickening
P13	Bilateral tubular and varicose bronchiectasis	None	Right inferior GGO; bilateral inferior intra- and interlobular thickening	None
P14	None	None	None	None
P15	None	None	Subpleural infiltrate	None

CT: Computed Tomography; HTLV-1: Human T lymphotropic virus type 1; P: patient; GGO: ground-glass opacities.
